# On the uneasy alliance between moral bioenhancement and utilitarianism

**DOI:** 10.1111/bioe.12974

**Published:** 2021-11-19

**Authors:** Karolina Kudlek

**Affiliations:** ^1^ Department of Philosophy University of Twente Enschede The Netherlands; ^2^ Institute of Philosophy Zagreb Croatia

**Keywords:** altruism, commonsense morality, impartiality, justice, moral bioenhancement, utilitarianism

## Abstract

Moral bioenhancement (MBE) is often associated with a consequentialist, especially utilitarian, framework, owing to its capacity to prevent great harm and motivate acts in accordance with basic moral principles such us universal impartial altruism or benevolence. However, it remains unclear whether we could de facto justify MBE on utilitarian grounds. This article examines whether there is a plausible utilitarian case for MBE and what the obstacles for justifying MBE on utilitarian grounds could be. More specifically, it explores the relationship between MBE and basic utilitarian principles, as well as its effects on utilitarian moral judgment. It seems that MBE could modify moral agents in ways that would accord with the main utilitarian demands and facilitate the adoption and realization of utilitarian prescriptions. Although MBE would, in principle, create preconditions for achieving utilitarian ends, I argue that there are certain limits to this claim. I identify and elaborate several ways in which MBE could undermine utilitarian moral judgment.

## 1 INTRODUCTION

Enhancements are often associated with a consequentialist framework. A tacit assumption in the debate seems to be that they would be overall optimific—that their benefits would outweigh the costs. For example, some authors suggest that the debate largely moves along standard consequentialist lines and often employs a utilitarian model of aggregation, usually some version of the rule‐utilitarian maximization principle.^1^ Others suggest that particular enhancements, such as moral bioenhancement (MBE), may even supplement utilitarian morality by motivating us to act in accordance with basic moral principles such as universal impartial altruism or benevolence.^2^ That is, the aim of MBE to improve dispositions to altruism and a sense of justice overlaps with the basic demands of utilitarian moral theory.^3^ Thus, MBE is discussed as a means not only of preventing great harm that threatens all the planet's inhabitants, but also of turning us into better utilitarian agents. However, in order to determine whether MBE is in fact justified on utilitarian grounds, we need to thoroughly examine its relationship with basic utilitarian principles, as well as its effects on moral agents and utilitarian moral judgment. Therefore, my goal here is to make a ruling on whether there is a plausible utilitarian case for MBE and to determine the potential obstacles to justifying MBE on utilitarian grounds.

MBE seems not merely permissible but also desirable on utilitarian grounds because it improves psychological dispositions which make people more likely to *act* like utilitarians. Utilitarianism is famous for demanding radical impartiality and strong benevolence in moral agents. It urges us to prevent bad things from happening whenever this is in our power.^4^ However, most people fail at adopting and following these principles owing to a lack of moral motivation, and ‘as many of us are incapable of complying even with commonsense morality, and the proposed consequentialist extension or revision of it is more demanding, it is obvious that compliance with this extended morality will require an enhancement of the moral motivation of many of us’.^5^ MBE promises to boost our moral motivation, making us more compliant with this extended morality, and helping us to deal with the pressing issues of the modern world.^6^ It is expected to modify our commonsense morality to the extent of counteracting the features of our moral psychology that are commonly associated with decisions resulting in bad outcomes, such as ‘the limitation of our altruism to those who are near and dear’ and ‘the numbness to larger numbers of sufferers’.^7^ To put this in utilitarian terms, it seems that MBE will promote good consequences by reinforcing the duties recognized by commonsense morality.^8^

In my attempt to explore the possible justifications of, and obstacles to, MBE on utilitarian grounds, I focus mainly on Persson and Savulescu's proposal for motivational MBE, which suggests improving particular features of commonsense morality.^9^ However, I consider the likely need for a more sophisticated kind of MBE—one that would encompass both motivational and deliberative capacities—and how such a possibility would resonate with utilitarian standards. My analysis consists of two main parts. First, I make a prima facie utilitarian case for MBE by looking at how MBE fits with basic utilitarian principles—such as impartiality and utility maximization. I examine whether MBE can make us better utilitarian agents and whether it is indeed optimific—such that it would yield greater benefits over costs. I show that MBE roughly meets basic utilitarian demands—enhancement of altruism and a sense of justice could contribute to the overall good. Second, I argue that MBE might not *always* be optimific, owing to limitations that we need to take into account. I argue that MBE's effects on utilitarian moral judgment would not be reliable and systematic but a matter of chance, and could, therefore, undermine the best utilitarian outcomes.

I look into three particular concerns: (a) enhancing commonsense morality may create tension between utilitarianism and MBE through fostering intuitive instead of rational reasoning; (b) enhancing altruism may promote partial instead of universal and impartial concern owing to the phenomenon of parochial altruism; (c) enhancing a sense of justice may lead to prioritizing just over utility‐maximizing actions. These concerns show that although there is, in principle, a legitimate case for MBE, it comes with certain limitations. As I will note, there may be ways to tackle these concerns, but the strength of these concerns will still depend on different accounts of utilitarianism and the kind of MBE applied (such as direct/indirect utilitarianism and motivational/deliberative MBE). My take is that direct utilitarians are unlikely to support MBE, whereas indirect utilitarians may have good reason to do so.

## 2 THE UTILITARIAN CASE FOR MBE

According to its proponents, MBE is expected to increase the likelihood that we correctly estimate the right thing to do and act upon it. However, the estimation of what constitutes the correct action will depend on personal beliefs and preferences:

To be morally enhanced is to have those dispositions which make it more likely that you will arrive at the correct judgement of what it is right to do and more likely to act on that judgement. It is disputed what the right thing to do is and how we would arrive at the right course of action. What constitutes moral enhancement will depend on the account one accepts of right action.^10^

In order to understand what this entails for utilitarian morality, we can start by examining whether the ends and means of MBE are right/permissible on utilitarian grounds. Thus, in this section, I will examine (i) how MBE affects moral agents and their actions (whether it promotes utilitarian ends), and (ii) whether the act of enhancement itself is right or permissible on utilitarian grounds (whether the means of MBE are acceptable). First, I look into MBE's correspondence with basic utilitarian principles and show that it could modify moral agents in ways that would *indirectly* facilitate utilitarian ends. Second, I explore the conditions that MBE would need to satisfy to be optimific, and I argue that there are good reasons to believe that it would meet these requirements.

### 2.1 Making better utilitarian agents?

Advocates of MBE envision this type of moral betterment as an extension of duties recognized by commonsense morality because such an approach may have the best overall consequences. ‘Folk’ or ‘commonsense’ morality is a globally shared set of moral attitudes that are ‘a common denominator of the diversely specified moralities of human societies over the world’.^11^ It amounts to ‘a set of psychological dispositions to react in particular ways in certain types of situations’.^12^ MBE is supposed to modify these dispositions. To fix some of the reoccurring flaws of moral psychology, Persson and Savulescu propose ‘a rather modest extension of commonsense morality, an extension which puts greater emphasis upon duties that commonsense morality already recognizes’.^13^ MBE is supposed to strengthen pro‐moral emotions (sympathy, cooperation, etc.) or, alternatively, diminish counter‐moral emotions (racial aversion, violent aggression, etc.).^14^

Although commonsense morality typically exhibits deontological features, Persson and Savulescu are confident that it will produce the best overall consequences:

For it may be that the structure of commonsense morality is so deeply embedded in our nature that it will have best consequences in terms of our underlying consequentialist theory if we try to live by something akin to commonsense morality, somewhat revised to be better aligned with the underlying consequentialist theory.^15^

They continue:

Many consequentialist theories nowadays have this sort of two‐level architecture: a ground level of true consequentialist principles—in our case about universal beneficence and justice—and a superstructure of literally false commonsensical principles, the endorsement of which is pragmatically justified by its good consequences, as determined by the ground‐level consequentialism.^16^

Let us grant this *arguendo*. It follows that MBE will, through the reinforcement of duties recognized by commonsense morality, likely promote an underlying consequentialism or the maximization of utility. Such an outcome seems to be in line with some kind of indirect utilitarianism (such as rule or motive utilitarianism), which instructs us to follow particular rules, motives, virtues, and so forth, because they have been shown to maximize utility in the long run.^17^ Compared with direct utilitarianism, indirect utilitarianism does not place so much normative weight on the outcomes of actions—rightness depends on whether the action follows from rules and dispositions of character that are themselves utility‐maximizing.^18^ For example, rule and motive utilitarianism determine an action's rightness by its conformity to the best set of rules or by the motivations with which it is performed.^19^ Since MBE amounts to improvements of the agent's character, it is more likely to be justified on indirect utilitarian grounds, whereas the rightness on direct (act) utilitarian grounds should be determined directly from the improvement's consequences.

Moreover, strengthening altruism and a sense of justice could facilitate the adoption of the standard utilitarian principles of impartiality and utility. MBE could broaden the scope of our moral concern, make us more likely to maximize utility, and less likely to harm others. By always urging us to maximize the welfare of everyone affected by our actions,^20^ utilitarianism presupposes strong altruistic and benevolent character traits in a moral agent or as Mill suggests: ‘[it] requires him to be as strictly impartial as a disinterested and benevolent spectator’.^21^ Mill also held that impartiality is an obligation of justice that can be influenced only by proper considerations (while resisting other motives). Impartiality is closely related to equality and, therefore, ‘one person's happiness (…) is counted for exactly as much as another's’.^22^ Hence, if MBE makes us more altruistic, impartial and just, it will create the necessary preconditions for facilitating utilitarian ends.

One of the MBE project's main agendas is to broaden our moral concern beyond the limits of kith and kin, so as to include those in need.^23^ A successful MBE is expected to override various biases that seem to be hard‐wired into our moral psychology (such as selfishness, nepotism, xenophobia, groupishness, etc.), which often cause us to behave in morally undesirable ways. The utilitarian principle of impartiality dictates precisely that ‘we broaden our concerns so that we are not focused just on ourselves, or on our friends, family or fellow citizens’ but on ‘everyone whose well‐being may be affected by our actions’.^24^ Hence, not only does MBE accord with the classic utilitarian principles of agent‐neutrality and impartiality, but it creates the grounds for promoting the kind of behaviour desired/favoured/advocated by utilitarians.

Utilitarian moral theory also gives weight to reducing pain and suffering of all sentient beings. One crucial aspect of MBE is preventing harm in terms of reducing crime and violent behaviour, which contributes to minimizing pain and suffering in the utilitarian sense. Preventing harm is also nothing but avoiding bad consequences, and MBE proponents deem that goal (including the means to achieve it) to be justified in a similar way to how utilitarians justify certain actions in terms of the goodness of outcomes. Thus, MBE can be understood as a means of preventing harm. In broad terms, utilitarianism requires us to adopt *any* means that will maximize the sum total of welfare in the world. If we can reasonably expect that the means of biomedical enhancement would maximize the sum total of welfare, there would be no reason for utilitarians to oppose enhancement. I will now explore whether MBE as an intervention could be justified on utilitarian grounds.

### 2.2 Is MBE optimific?

Aside from promoting desirable dispositions of character, utilitarians might be interested in how MBEs score on the utilitarian calculus. Generally, if an action is to be morally justified on utilitarian grounds, it must be optimific—such as to yield the greatest balance of benefits over costs.^25^ In other words, the rightness of actions is explained in terms of the goodness of outcomes. For example, act consequentialists hold that an action is right if it results in an outcome that is ‘at least as good as that of any relevant alternative’.^26^ Utilitarians hold that one outcome is better than another if it contains more well‐being. Thus, MBE would be right on utilitarian grounds when it creates more (or no less) well‐being than any available alternative. In terms of an agent's well‐being, MBE is good insofar as it promotes value within an agent's life and bad insofar as it inhibits it. But we want to determine here whether MBE itself or *on the whole* is the best possible action compared with its alternatives.

Commonly discussed alternatives to MBE are, for example, the preservation of the status quo, traditional moral enhancement, and cognitive enhancement.^27^ And although MBE faces fierce criticism, one could argue that the currently conceivable alternatives are not likely to score any better on the utilitarian calculus. Following basic utilitarian rationales, MBE would be morally right if we could reasonably expect its outcomes to be optimific, that is, that none of the available alternatives results in a larger sum total of welfare in the world. The ultimate motive for implementing MBE is not the quest for perfection, but the urgent need to deal with the pressing issues of the modern world, such as environmental degradation and threats of terrorist attacks.^28^ Hence, the right action has to produce the greatest balance of benefits over costs to urgently prevent catastrophic harm, in view of the lack of moral motivation and immoral behaviour.

The preservation of the status quo is often advocated by bioconservative authors who believe that maintaining things as they are is better and safer than pursuing new and unfamiliar projects such as MBE.^29^ New projects are often risky, controversial, and may not be worth our while—or in this context, the risks might outweigh the benefits. This view also, to some extent, implies that the quality of our lives is satisfactory as it is which is not obviously true. Biomedical enhancements might become necessary for *sustaining* the status quo rather than improving our situation—to prevent a decline in our average quality of life.^30^ The decline in life quality (such as environmental degradation) cannot be prevented by mere inaction. In other words, sticking to the status quo is likely to make matters worse, and will surely inhibit our well‐being.

Traditional moral enhancement resembles the preservation of the status quo, since it amounts to already established, noninvasive means of moral improvement. Although considerable moral progress has been achieved throughout history, traditional moral enhancement works too slowly and is, therefore, insufficient in the face of the need for urgent solutions.^31^ For traditional moral enhancement methods to be sufficient, we would already need to be morally motivated to a significant extent.^32^ Even if they were significantly more advanced than they currently are, the traditional means (such as moral education, upbringing, and socialization) would hardly be successful in boosting our motivation for dealing with pressing issues, or in preventing harm on a larger scale. Thus, traditional moral enhancement is unlikely to result in outcomes better than those expected by MBE.

Cognitive enhancement might have a more immediate effect on gaining the relevant knowledge and skills to deal with pressing issues. However, ‘we do not necessarily do what is right and good as soon as we gain knowledge of what this is’.^33^ Some aspects of cognitive enhancement might be necessary as one ingredient of effective moral enhancement, but it is not likely that cognitive enhancement would be sufficient on its own for reaching morally desirable improvement. Most importantly, the risk of abuse appears to be much greater with cognitive enhancement than with MBE because the former directly benefits the enhanced, but may disadvantage the unenhanced.^34^ MBE, on the other hand, is expected to largely benefit the unenhanced.^35^

Insofar as there is no better alternative for meeting pressing issues, MBE could be optimific on the utilitarian calculus. This analysis takes the feasibility of MBE at face value and presupposes it would achieve its stated goals. But even if it had negative side‐effects, such as harming the moral agent by undermining their freedom or autonomy, MBE may still be optimific. This is because utilitarianism places no constraints on the maximization of utility—it permits us to cause harm for the sake of maximizing utility. Moreover, if MBE is optimific, and thus right from the utilitarian point of view, its implementation is required: not conducting the right action counts as moral wrongdoing on utilitarian grounds.^36^

Thus far, I have identified some conditions under which MBE would be justified on utilitarian grounds. It seems not only that MBE is in line with basic utilitarian principles, but also that it could indirectly maximize utility by modifying certain dispositions of character. Also, insofar as MBE is our best bet (optimific) for solving the pressing issues of the modern world, utilitarians should fully support its implementation. However, there might be some limits to these claims. In the following section, I raise several concerns and identify conditions under which MBE could undermine utilitarian judgment.

## 3 THE UTILITARIAN CASE AGAINST MBE

The motivational model of MBE that I have described may have effects on moral agents and their judgment that could be problematic from the utilitarian point of view. I will address three such concerns in this section. Some of these concerns have been acknowledged in the broader debate, but their implications for utilitarian morality are yet to be discussed. I start from some conceptual difficulties regarding utilitarianism and commonsense morality, which can create tension between utilitarianism and MBE. I then examine how specifically the enhancement of altruism and a sense of justice could conflict with utilitarian demands for impartiality and the maximization of utility. I also take notice of how these concerns could be mitigated.

### 3.1 The tension between utilitarianism and commonsense morality

One possible source of tension between MBE and utilitarianism is their arguably different relation to commonsense morality. Namely, MBE tends to extend parts of commonsense morality (as explained previously), whereas utilitarianism departs considerably from this moral framework. If so, it follows that MBE departs from utilitarianism, and should therefore be alarming to utilitarians. I noted at the beginning that MBE is conceived of as a modest extension of the globally shared set of moral attitudes that are based in biology and often referred to as folk or commonsense morality.^37^ On this view, commonsense morality amounts to a set of psychological dispositions for reacting in certain ways^38^; MBE would boost either these dispositions, or the commonly shared intuitions about what is the morally right thing to do.

Conversely, utilitarianism is typically regarded as a view rooted in rational reflection and in conflict with many commonsensical intuitions. The strongest opposition to utilitarianism is, in fact, due to its clash with common moral judgment and intuitions.^39^ For example, Kahane et al. describe two ways in which utilitarianism radically departs from commonsense morality: utilitarianism (i) places no constraints on the maximization of aggregate well‐being, and (ii) requires us to maximize the well‐being of all sentient beings.^40^ By placing no constraints, utilitarianism permits us to harm others for the sake of maximizing well‐being. Such reasoning often conflicts with commonsense intuitions. Hand in hand with counter‐intuitiveness goes the problem of demandingness. By requiring a universal and impartial maximization of well‐being, utilitarianism strikes many as too demanding.^41^ Thus, utilitarianism can permit too much and demand too much, and is, therefore, in conflict with our common moral judgments. Unlike utilitarian views, common moral views have their source in gut reactions and intuitions shaped by evolutionary pressures.^42^

Biologically based moral attitudes, which are altered via MBE, are often regarded as quick intuitive judgments in moral psychology. Such intuitive judgments (typical for commonsense moral reasoning) often lead to conclusions favoured by deontological and rights‐based doctrines, whereas careful rational reflection has been shown to produce more utilitarian‐friendly conclusions.^43^ Assuming that utilitarianism radically departs from commonsense morality and that MBE is conceived of as extending/building upon the latter, it follows that MBE radically departs from utilitarianism. If MBE fosters commonsensical reasoning, utilitarians, who tend to prefer a radically rational decision‐making approach, could rightfully doubt whether such an intervention is overall optimific.

This concern may be mitigated. For example, it has been argued that motivational MBE will have to be supplemented by the enhancement of deliberative capacities (cognitive enhancement) in order to achieve the desired aims.^44^ If this were the case, we might expect a more balanced approach to enhancing moral decision‐making that would not necessarily undermine rational deliberation.^45^ Hence, as far as MBE fosters intuitive thinking, utilitarians would find it problematic. But even under these circumstances, not all utilitarians need to think alike. Another possibility is to resort to the previously discussed view that commonsense morality is de facto consequentially justified on *indirect* utilitarian grounds because (as I show in Section 2) it would boost behavioural tendencies that maximize well‐being in the long run.^46^ If commonsense moral tendencies can be justified on utilitarian grounds, then there was no conflict between commonsense morality and utilitarianism to begin with. Instead, they may form some sort of friendly alliance.^47^ I, however, believe that the gap between commonsense morality and the utilitarian doctrine is further reflected in the practical aspects of enhancing altruism and a sense of justice.

### 3.2 The problem of partial altruism

On a more practical level, enhancing the psychological disposition to altruism could undermine the impartial maximization of utility due to the natural tendency towards partial or parochial altruism. Although this concern has been noted in the bioenhancement debate,^48^ it has not been previously raised in discussions about utilitarianism. My goal here is to examine the implications of partial altruism for utilitarian morality. To have an attitude of altruism and sympathy towards other beings is to want things to go well for them for their own sake.^49^ Enhancing the capacity for sympathy would then mean enhancing the probability that we will do what we believe we ought to do (in response to reasons for it). In this sense, MBE can be interpreted as a suggestion to make human beings, on average, more prone to altruism and sympathy; namely, to the same extent as the most moral people among us already are. In practice, this would mean that a person with enhanced altruism will have a stronger tendency to perform altruistic acts. For example, a person who already gives money to charity will now give even more. Insofar as we believe that giving to charities is optimific, but that there are psychological constraints on our ability to sacrifice our well‐being for the sake of others,^50^ MBE can bring us closer to the utilitarian ideal.

However, since the natural attitude of sympathy tends to be partial, it may conflict with utilitarian demands for impartiality:

It is well‐known that the attitude of sympathy, as it occurs spontaneously, tends to be partial: we tend to sympathize in particular with members of our family, friends, and people before our eyes. (…) Utilitarianism, which takes sympathy or altruism to be the one and only fundamental moral attitude, opposes this partiality, by declaring in its most familiar form, roughly speaking, our moral goal to be to see to it that things go as well as possible for as many as possible.^51^

It turns out that we naturally tend to sympathize *more* with members of our in‐group such as friends and family, while our willingness to cooperate or sympathize with out‐group members tends to be reduced and hostility towards them increased.^52^ Increasing altruistic emotions in human beings in order to increase the probability that one does what one believes to be right could, therefore, *further* strengthen in‐group, instead of out‐group, altruism. Such side‐effects were reported by several studies about the administration of oxytocin and serotonin, which are typically expected to increase altruistic tendencies in humans.^53^ Hence, strengthening our psychological disposition to altruism could indeed promote moral concern for members of our close group, but the concern may not necessarily become more extended beyond that group.

By virtue of the phenomenon of partial (or parochial) altruism, the effects of MBE might be limited in scope, and utilitarians might find this troubling. Since utilitarianism places significant normative weight on agent neutrality, enhancement of altruism could, under some circumstances, undermine the principle of impartiality. For example, parents with enhanced altruism might prefer marginal increases in their children's well‐being over massive increases in the well‐being of a great number of other children.^54^ As long as the enhancement of altruism promotes only in‐group cooperation and sympathy, its potential to prompt utilitarian judgments among agents will be limited.

However, promoting in‐group altruism can be consistent with utilitarianism when it maximizes the overall good for all affected parties. Utilitarians will sometimes judge MBE to have morally desirable effects even when the consequences of actions are strictly limited to one's group. Imagine a negligent parent who undergoes enhancement to become more altruistic towards her own children. In this case, the enhancement of ‘partialist’ altruism would be compatible with utilitarian demands because all affected parties belong to one's group. Also, if it turned out to be possible for MBE to go ‘hand in hand with reasoning which undercuts race, sex etc. as grounds for moral differentiation’,^55^ then this concern would be alleviated. Another solution could be a distinction between ‘utilitarian’ and ‘utile’, where utile implies doing more good for others even if it is a restricted set of others. Apart from cases where utilitarianism allows certain forms of partiality, the enhancement of altruism would not necessarily facilitate utilitarian ends.

### 3.3 Prioritizing just over utility‐maximizing actions

To solve the problem of partialist altruism and to supplement the principle of utility with some principle of justice, Persson and Savulescu postulate another moral attitude—a sense of justice or fairness in moral agents.^56^ As a moral theory, utilitarianism is often criticized for being unable to take matters of justice into proper account,^57^ since maximizing overall well‐being may sometimes strike us as unjust or unfair.^58^ Therefore, ‘the moral goal is not just that things go as well as possible for as many as possible, but that how well things go for different beings be as much as possible in line with justice’.^59^ In short, MBE is expected to ‘motivate us to act in accordance with plausible basic moral principles’,^60^ such as impartial altruism and benevolence, which happen to overlap with basic utilitarian principles.

But justice is an exceptionally complex and controversial notion, and there is little consensus on what it consists of. I will understand it here, following Persson and Savulescu's explanation, as a desire to do what one thinks is just or fair, which, in turn, makes the enhancement of one's sense of justice simply the enhancement of one's desire (or the motivation and probability) to do what one regards as just.^61^ Authors acknowledge that our common sense is ‘firmly wedded to deserts and rights’, and that our sense of justice originates in reciprocity and tit‐for‐tat strategies: justice requires getting what you deserve.^62^ Thus, instead of balancing out the partial altruism problem, the enhancement of a sense of justice can create additional problems for utilitarians.

As with altruism, the biological sense of justice does not necessarily align with the utilitarian one. According to the utilitarian conception of justice, just actions are those that maximize utility. For example, the justice of institutions requires that institutions maximize utility.^63^ I mentioned previously that most people are not naturally inclined to utilitarian moral reasoning—we often find it counterintuitive and unjust. Thus, one could argue that a moral attitude that is supposed to ensure that things are in line with justice can, in some circumstances, undermine or conflict with the willingness to maximize utility. For example, we often have difficulty sacrificing an innocent person's well‐being for the sake of maximizing utility. If you have a strong intuition that it is not fair to sacrifice one for the sake of many, boosting your sense of justice could only deepen this inclination. Hence, boosting a biological sense of justice and fairness (i.e., enhancing the desire to do what we regard as just) might reduce the likelihood of arriving at the correct utilitarian judgment, as well as of acting upon it.

Even if utilitarians conceive of the ‘good’ and the ‘just’ independently from one another, these values may conflict given that it is not always possible to maximize the good and act justly at the same time. Sometimes you have to lie to your friend to make them feel better. In such cases, act utilitarians are required to always maximize the good, even when that means committing unjust acts. Indirect utilitarians, however, acknowledge that, although utility is the ultimate foundation for morality, the direct pursuit of utility is not always the best course of action.^64^ Therefore, they conform to the rule that lying does not pay off overall. Now, an enhanced sense of justice would entail a stronger inclination to do what we deem just, regardless of what directly maximizes utility. This means that enhanced agents will be more likely to follow indirect than direct utilitarian prescriptions. In other words, MBE is more likely to be justified on indirect than on direct utilitarian grounds.

An enhanced sense of justice can accord with utilitarian demands in a limited capacity or when supposedly just acts have been shown to maximize overall utility. But this would be merely a matter of chance—there is no direct connection between MBE and utilitarian outcomes. In other cases, MBE can interfere with maximizing utility—it can make us favour just over utility‐maximizing acts (assuming that the notion of justice does not coincide with maximum goodness). An enhanced sense of justice can also interfere with the utilitarian demand for altruism—it can make us favour just over altruistic acts (when altruism would be preferable from a utilitarian perspective). A simultaneous enhancement of a sense of justice along with the capacity for altruism will not resolve any of these issues. On the contrary, it may only deepen them because moral agents will experience stronger preferences or a stronger conflict of preferences.

A remedy to these concerns could once again lie in the further development of technology that would fine‐tune the enhancement of motivational and deliberative capacities. If the technology became sophisticated enough, some of the aforementioned problems could be avoided. Whether this is indeed possible is, however, a separate question. From where we are standing now, tampering with parts of commonsense morality (specifically the capacities for altruism and justice) may, under the described circumstances, compromise reaching the best utilitarian judgment. How strict these limitations will be will greatly depend, as already mentioned, on the different notions of utilitarianism and different approaches to MBE. But it seems safe to conclude that direct utilitarians are less likely to support MBE, whereas indirect utilitarians may have good reasons to do so.

## 4 CONCLUSION

My main goal here was to explore whether there are legitimate reasons to think about MBE as justified on utilitarian grounds. The analysis showed that there is indeed a prima facie case that MBE would be appealing to utilitarians. It seems to roughly accord with utilitarian demands and principles, especially in terms of expanding moral concern and adopting agent neutrality. We could deem MBE optimific as far as it is our best solution to urgently prevent harm caused by the lack of moral motivation and immoral behaviour. However, there are important limits to this claim. I argued that one such constraint is the well‐known tension between utilitarianism and commonsense morality. Since utilitarianism places great normative weight on radically rational mode of decision‐making, it might not favour enhancing the quick intuitive judgments typical for commonsensical reasoning. This problem is further reflected in the potential to promote partial altruism and prioritize just over utility‐maximizing actions, which could compromise utilitarian moral judgment. Although these concerns may place tight constraints on justifying MBE on direct utilitarian grounds, they apply with less strength in the case of indirect utilitarianism and (as of yet hypothetical) sophisticated types of MBE, such as the fine‐tuned enhancement of motivational and deliberative capacities.

## Footnotes

1 This is, for example, discussed in Heinrichs, J. H., & Stake, M. (2018). Enhancement: Consequentialist arguments. *Zeitschrift für Ethik und Moralphilosophie*, *1*, 321–342. For a broader discussion on how ethical theories can provide guidance for the evaluation of human enhancement technologies, see Kudlek, K. (forthcoming). Challenges in the human enhancement debate: a critical review. *Techné: Research in Philosophy and Technology*, 26: 2; and for a related discussion on the supposed clash between MBE and deontological values, see Kudlek, K., & Smith, P.T. (forthcoming). The Kantian promise and peril of moral bioenhancement. *Journal of Applied Philosophy*.

2 Persson, I., & Savulescu, J. (2015). Reply to commentators on *Unfit for the future. Journal of Medical Ethics*, *41*(4), 348–352, p. 349.

3 Ibid.

4 Singer, P. (1972). Famine, affluence and morality. *Philosophy and Public Affairs*, *1*(3), 229–243.

5 Persson & Savulescu, op. cit. note 2.

6 Persson, I., & Savulescu, J. (2012). *Unfit for the future: The need for moral enhancement*. Oxford, U.K.: Oxford University Press.

7 Ibid: 123.

8 Persson & Savulescu, op. cit. note 2.

9 I also take the feasibility of this proposal at face value; that is, I do not discuss whether such enhancement is possible, but how we should morally evaluate it if it were possible.

10 Savulescu, J., & Persson, I. (2012). Moral enhancement, freedom, and the god machine. *Monist*, *95*(3), 399–421.

11 Persson & Savulescu, op. cit. note 6, p. 12.

12 Gligorov, N. (2018). The cognitive basis of commonsense morality. *Journal of Cognitive Enhancement*, *2*, 369–376, p. 370.

13 Persson & Savulescu, op. cit. note 6, p. 123.

14 Douglas, T. (2008). Moral enhancement. *Journal of Applied Philosophy*, *25*(3), 228–245. Although diminishing counter‐moral emotions is as relevant as boosting pro‐moral emotions, I will limit my analysis to the latter. One reason is the strong utilitarian focus on *active* contribution to overall well‐being.

15 Persson & Savulescu, op. cit. note 2, p. 349.

16 Ibid.

17 Brandt, R. (1992). *Morality, utilitarianism, and rights*. Cambridge, U.K.: Cambridge University Press; Sverdlik, S. (2011). *Motives and rightness*. Oxford, U.K.: Oxford University Press.

18 Alexander, L. (1985). Pursuing the good‐indirectly. *Ethics*, *95*(2), 315–332.

19 Woodard, C. (2019). *Taking utilitarianism seriously*. Oxford, U.K.: Oxford University Press.

20 Bentham, J. (2000). *An introduction to the principles of morals and legislation*. Kitchener, Ont: Batoche, pp. 14‐15; Mill, J. S. (1864). *Utilitarianism* (2nd ed.). London: Longman, Green, Longman, Roberts, and Green.

21 Mill, op. cit. note 20, p. 24.

22 Ibid: 93.

23 Persson & Savulescu, op. cit. note 6.

24 Shafer‐Landau, R. (2012). *The fundamentals of ethics*. Oxford, U.K.: Oxford University Press, p. 125.

25 Rachels, J. (2003). *The elements of moral philosophy*. New York, NY: McGraw‐Hill.

26 Woodard, op. cit. note 19, p. 4.

27 This is not an exhaustive list of alternatives to pressing issues in the enhancement debate. However, the listed possibilities are among those most commonly discussed. For a more detailed discussion on these topics, see Sandel, M. J. (2007). *The case against perfection: Ethics in the age of genetic engineering*. Cambridge, MA: Harvard University Press; Buchanan, A. (2011). *Beyond humanity? The ethics of biomedical enhancement*. Oxford, U.K.: Oxford University Press, chapter 3; Sandberg, A. (2011). Cognition enhancement—Upgrading the brain. In J. Savulescu, R. ter Meulen, & G. Kahane (Eds.). *Enhancing human capacities* (pp. 71–91). Oxford, U.K.: Wiley‐Blackwell.

28 Persson & Savulescu, op. cit. note 6.

29 For instance, Sandel, op. cit. note 27; Kass, L. (2003). Ageless bodies, happy souls: Biotechnology and the pursuit of perfection. *New Atlantis*, *1*, 9–28. Nevertheless, mentioned bioconservatives are not only concerned with enhancement's bad outcomes but also believe there may be something *intrinsically* wrong with them. I discuss this possibility in detail in Kudlek, K. (2021). Is human enhancement intrinsically bad? *Medicine, Health Care and Philosophy*, 24, 269‐279.

30 Buchanan, op. cit. note 27, p. 76.

31 Persson & Savulescu, op. cit. note 6.

32 Persson, I., & Savulescu, J. (2008). The perils of cognitive enhancement and the urgent imperative to enhance the moral character of humanity. *Journal of Applied Philosophy*, *25*(3), 162–177, p. 168.

33 Ibid: 168.

34 This primarily applies to competitive environments such as sports and work, in which cognitive enhancements are associated with positional goods—goods whose value is dependent on others not having them (Sandberg, op. cit. note 27, p. 83).

35 Douglas, op. cit. note 14, p. 230.

36 Shafer‐Landau, op. cit. note 24.

37 Persson & Savulescu, op. cit. note 6.

38 Gligorov, op. cit. note 12.

39 Singer, P. (2005). Ethics and intuitions. *Journal of Ethics*, *9*(3–4), 331–352; Rachels, J. (1991). When philosophers shoot from the hip. *Bioethics*, *5*, 67–71.

40 Kahane, G., Everett, J. A. C., Earp, B. D., Caviola, L., Faber, N. S., Crockett, M. J., & Savulescu, J. (2018). Beyond sacrificial harm: A two‐dimensional model of utilitarian psychology. *Psychological Review*, *125*(2), 131–164, p. 132.

41 Kagan, S. (1984). Does consequentialism demand too much? Recent work on the limits of obligation. *Philosophy & Public Affairs*, *13*(3), 239–254; Cullity, G. (2003). Asking too much. *Monist*, *86*(3), 402–418.

42 Kahane et al., op. cit. note 40.

43 Haidt, J. (2012). *The righteous mind: Why good people are divided by politics and religion*. New York, NY: Vintage; Greene, J. D. (2007). Why are VMPFC patients more utilitarian? A dual‐process theory of moral judgment explains. *Trends in Cognitive Sciences*, *11*, 322–323; Paxton, J. M., Bruni, T., & Greene, J. D. (2014). Are ‘counterintuitive’ deontological judgments really counterintuitive? An empirical reply to Kahane et al. (2012). *Social Cognitive and Affective Neuroscience*, *9*, 1368–1371.

44 For instance, Persson & Savulescu, op. cit. note 6 or Earp, B. D., Douglas, T., & Savulescu, J. (2017). Moral neuroenhancement. In S. Johnson & K. Rommelfanger (Eds.), *Routledge handbook of neuroethics* (pp. 166–184). Abingdon, U.K.: Routledge.

45 Although the discussion has recognized that motivational enhancement might not be sufficient to achieve the desired moral betterment on its own, it is still discussed as a legitimate ‘standalone’ type of MBE. One reason for this might be that a more sophisticated MBE may be overly speculative at this point. In addition, the distinction between direct and indirect MBE captures the difference between motivational and deliberative MBE to some extent; see, e.g., Schaefer, G. O. (2015). Direct vs. indirect moral enhancement. *Kennedy Institute of Ethics Journal*, *25*(3), 261–289; Earp et al., op. cit. note 44.

46 Even if MBE can be justified on indirect utilitarian grounds, it could still be problematic from a direct utilitarian perspective. Act (outcome) utilitarians would evaluate MBE by its immediate consequences.

47 In philosophical theory, the relationship between utilitarianism and commonsense morality can be seen as a friendly alliance because commonly accepted moral rules (often referred to as ‘rules of thumb') usually tend to maximize happiness/utility (Sidgwick, H. (1907). *The methods of ethics*. London: MacMillan). In the context of MBE, however, a biological approach to commonsense morality (which implies their conflict) is more relevant and, therefore, given more attention in this discussion. Regardless of whether the relationship between utilitarianism and commonsense morality should be characterized as one of alliance or dissent, MBE (extending upon commonsense morality) can cause problems for utilitarians. For some views about how MBE could disrupt the valuable balance between commonsense morality and utilitarianism, see Agar, N. (2015). Moral bioenhancement and the utilitarian catastrophe. *Cambridge Quarterly of Healthcare Ethics*, *24*, 37–47.

48 Persson & Savulescu, op. cit. note 6.

49 Persson, I., & Savulescu, J. (2018). The moral importance of reflective empathy. *Neuroethics*, *11*, 183–193.

50 Singer, op. cit. note 4.

51 Persson, I., & Savulescu, J. (2019). The duty to be morally enhanced. *Topoi*, *38*, 7–14, p. 8.

52 For a more detailed account of this concern see, for example, Kudlek, K. (2019). The role of emotion modulation in moral bioenhancement debate. *Topoi*, *38*, 113–123.

53 See, for example, De Dreu, C. K. W., Greer, L. L., Handgraaf, M. J. J., Shalvi, S., Van Kleef, G. A., Baas, M., … Feith, S. W. W. (2010). The neuropeptide oxytocin regulates parochial altruism in intergroup conflict among humans. *Science*, *328*, 1408–1411; De Dreu, C. K. W. (2011). Oxytocin promotes human ethnocentrism. *Proceedings of the National Academy of Sciences of the United States of America*, *108*, 1262–1266; Crockett, M. J., Apergis‐Schoute, A., Herrmann, B., Lieberman, M. D., Müller, U., Robbins, T. W., & Clark, L. (2013). Serotonin modulates striatal responses to fairness and retaliation in humans. *Journal of Neuroscience*, *33*(8), 3505–3513.

54 A similar example is offered in: Drake, N. (2016). Is moral bioenhancement dangerous? *Journal of Medical Ethics*, *42*(1), 3–6.

55 Persson & Savulescu, op. cit. note 6, p. 120.

56 Persson & Savulescu, op. cit. note 2, p. 349.

57 Ibid.

58 Persson & Savulescu, op. cit. note 51, p. 8.

59 Ibid.

60 Persson & Savulescu, op. cit. note 2, p. 349.

61 Persson & Savulescu, op. cit. note 51.

62 Ibid.

63 The lack of a plausible distinction between the notions of justice and goodness is a common problem for utilitarian moral theory. For an in‐depth discussion on this issue, see Woodard, op. cit. note 19, chapter 7.

64 Alexander, op. cit. note 18, p. 316.

